# Beat Keeping in a Sea Lion As Coupled Oscillation: Implications for Comparative Understanding of Human Rhythm

**DOI:** 10.3389/fnins.2016.00257

**Published:** 2016-06-03

**Authors:** Andrew A. Rouse, Peter F. Cook, Edward W. Large, Colleen Reichmuth

**Affiliations:** ^1^Long Marine Laboratory, Institute of Marine Sciences, University of California Santa CruzSanta Cruz, CA, USA; ^2^Department of Psychology, Emory UniversityAtlanta, GA, USA; ^3^Department of Psychological Sciences, University of ConnecticutStorrs, CT, USA

**Keywords:** sensorimotor synchronization, rhythmic entrainment, neural oscillators, sea lions, music cognition and perception, non-human models

## Abstract

Human capacity for entraining movement to external rhythms—i.e., beat keeping—is ubiquitous, but its evolutionary history and neural underpinnings remain a mystery. Recent findings of entrainment to simple and complex rhythms in non-human animals pave the way for a novel comparative approach to assess the origins and mechanisms of rhythmic behavior. The most reliable non-human beat keeper to date is a California sea lion, Ronan, who was trained to match head movements to isochronous repeating stimuli and showed spontaneous generalization of this ability to novel tempos and to the complex rhythms of music. Does Ronan's performance rely on the same neural mechanisms as human rhythmic behavior? In the current study, we presented Ronan with simple rhythmic stimuli at novel tempos. On some trials, we introduced “perturbations,” altering either tempo or phase in the middle of a presentation. Ronan quickly adjusted her behavior following all perturbations, recovering her consistent phase and tempo relationships to the stimulus within a few beats. Ronan's performance was consistent with predictions of mathematical models describing coupled oscillation: a model relying solely on phase coupling strongly matched her behavior, and the model was further improved with the addition of period coupling. These findings are the clearest evidence yet for parity in human and non-human beat keeping and support the view that the human ability to perceive and move in time to rhythm may be rooted in broadly conserved neural mechanisms.

## Introduction

Auditory-motoric entrainment—the coordination of motor movement with simple and complex rhythmic sounds—has a strong presence in human culture and is found across all human societies (Clayton et al., 2005). This phenomenon of “beat keeping” was believed to be unique to humans (Wallin et al., 2000; Bispham, 2006; Zatorre et al., 2007), but new findings in non-human animals have decisively put that idea to rest. Evidence for some faculty to flexibly entrain movement to simple metronome-like stimuli has been found in bonobos (Large and Gray, 2015), chimpanzees (Hattori et al., 2013), and budgerigars (Hasegawa et al., 2011). The ability to entrain to more complex musical stimuli has been shown in cockatoos (Patel et al., 2009), parrots (Schachner et al., 2009), and most reliably, a California sea lion (Cook et al., 2013). Preliminary evidence suggesting beat-keeping behavior has also been identified for elephants (Schachner et al., 2009) and horses (Bregman et al., 2013). While hypotheses have been advanced suggesting that beat keeping is dependent on specialized and relatively rare neural adaptations (Patel et al., 2009; Merchant and Honing, 2013), or exposure to auditory rhythm during critical developmental periods (Schachner, 2012), it is increasingly difficult to identify candidate traits exclusive to the phylogenetically distant species now shown capable of rhythmic entrainment. This suggests that rather than being a derived ability, this faculty is instead broadly conserved, supported by mechanisms of domain-general sensorimotor synchronization found across the animal kingdom (see Wilson and Cook, 2016).

Performance dynamics and variability in human rhythmic behavior have been extensively and carefully studied (see Repp, 2005; Large, 2008; Repp and Su, 2013 for reviews). Although beat-keeping behavior has now been ascribed to a number of non-human species, the mechanisms have not yet been explored outside of humans. If human rhythm is broadly conserved, beat keeping in other animals should be consistent with the principles governing the behavior in humans. One parsimonious and well-established theory of beat keeping is that of neural resonance. This theory proposes that the perception of pulse in simple and complex rhythms, and associated behavioral synchronization to those rhythms, arise from intrinsic properties of neural oscillation (Large and Snyder, 2009; Large et al., 2015). Unlike information-processing theories, in which beat perception and synchronization are separate computational processes that require specialized neural circuitry (Vorberg and Wing, 1996; Repp and Keller, 2004; Patel, 2006; Patel and Iversen, 2014), the theory of neural resonance states that both phenomena are byproducts of the physical principles of coupled oscillation (Large, 2008), and does not presuppose any specialized and potentially restricted neural adaptations beyond auditory-motor coupling to explain auditory motor entrainment.

Neural resonance theory is supported by the well-established finding of neural oscillation: interaction between excitatory and inhibitory neuronal populations gives rise to population rhythms throughout the brain, including across sensory and motor networks (Brunel, 2003; Börgers and Kopell, 2003; Buzsáki and Draughn, 2004; Stefanescu and Jirsa, 2008). In brief, when acoustic stimuli are presented in a periodic pattern, auditory oscillations spontaneously entrain to the structure of the stimulus stream (Will and Berg, 2007; Nozaradan et al., 2011, 2012). Presumably, these auditory oscillations then induce synchronized neural oscillations in coupled motor systems, leading to rhythmic behavior with a strong phase and tempo relationship to the auditory stimulus (e.g., Loehr et al., 2011). Models of neural resonance are neurologically plausible and fully compatible with widely accepted models of functional connectivity in the brain (see Biswal et al., 1995). The brain can be described as a complicated set of overlapping networks linking neural populations into functional units (Bullmore and Sporns, 2009), and connectivity between these units can be described in terms of synchrony of firing rates between neural populations (Biswal et al., 1995; Greicius et al., 2003). Perception and cognition are then posited to emerge out of the action and interaction of these networks (Sporns et al., 2004; Bressler and Menon, 2010). Importantly, although neural resonance does not require specialized neural mechanisms beyond linked auditory and motor networks, beat-keeping behavior is not necessarily obligate and automatic. Learning clearly changes the properties of coupling between auditory and motor networks, and attention and intention play important roles in producing or inhibiting beat-keeping behavior (Large and Jones, 1999; Repp and Keller, 2004).

An advantage of this sort of theoretical analysis is the ability to link complex oscillation of high dimensional neuronal populations with simpler lower dimensional population- and behavior-level models that capture much of the behavioral richness observed in high dimensional systems (Wilson and Cowan, 1973; Stefanescu and Jirsa, 2008) and that are amenable to theoretical and computational analysis (Aronson et al., 1990; Hoppensteadt and Izhikevich, 1996).

Neural resonance models have been used extensively to accurately describe rhythmic entrainment in humans, for both simple and complex stimuli. A common experimental approach uses behavioral paradigms that involve perturbations in both phase and tempo (Michon, 1967; Large and Palmer, 2002; Large et al., 2002; Repp and Keller, 2004; Loehr et al., 2011). Animal synchronization studies have historically attempted to demonstrate synchronization using statistical methods designed to show a nonrandom phase relationship between stimulus and movement (e.g., Patel et al., 2009, see Fisher, 1993; Pikovsky et al., 2001). However, to probe the underlying mechanisms, a different approach is required. One avenue is to perturb the stimulus and observe relaxation back to steady state behavior (a stable phase relationship).

Behavioral responses to stimulus perturbations can be modeled using the same discrete-time model of coupled oscillation that has been applied to both perception of rhythmic auditory sequences (Large and Jones, 1999) and perception-action coordination with rhythmic auditory sequences (deGuzman and Kelso, 1991; Loehr et al., 2011). Conceptually, a neural/behavioral oscillation is coupled to a rhythmic auditory stimulus that consists of brief acoustic events. The model assumes that the behavioral oscillation is temporally continuous, and the stimulus sequence is temporally discrete, illustrated in Equation (1).

(1) $$\begin{array}{l} \phi_{n+1}=\phi_{n}+\Omega -\alpha \text{ sin }\phi_{n} \end{array}$$

Here, ϕ_*n*_ is the phase of the behavioral oscillation at which acoustic event *n* occurs. The model predicts the phase of the behavioral oscillation ϕ_*n*+1_ at the next acoustic event, given the relative frequency of the behavioral oscillation and stimulus, Ω = 2π*f*_*osc*_∕*f*_*stim*_, and the coupling between the two, −α sin ϕ_*n*_. Generic models such as Equation (1) are particularly powerful because they make strong predictions regarding both steady state (synchronization) and transient (relaxation) behavior and are easily implemented and analyzed. However, they have not yet been applied to examine rhythmic behavior in non-human animals.

The most reliable and precise non-human beat keeper known is Ronan, a California sea lion (*Zalophus californianus*) who was trained using operant methods to match her head movement to a simple isochronous stimulus, thus “bobbing” her head in time to the rhythmic beats. Once she had learned to bob in time to simple stimuli at set tempos, she successfully transferred to novel tempos and stimuli, including music at multiple tempos (Cook et al., 2013). To date, Ronan's results represent the most extensive and robust dataset of beat keeping in a non-human animal. This sea lion's ability to entrain her body movement to sound makes her a valuable candidate for cross-species testing of theories and allows the examination of the underlying mechanisms in a broader comparative approach.

Here, we apply neural resonance theory to an experimental study of Ronan's entrainment behavior, in which we tested her ability to adapt to sudden changes in both the phase and tempo of an isochronous repeating stimulus. Although—like humans—Ronan had previously shown strong entrainment to complex musical stimuli, we used simple stimuli comparable to those presented to humans in similar studies. To determine whether her performance was consistent with theories of neural resonance as is seen in humans, we evaluated her performance with a discrete-time model of coupled oscillation (see Equation 1). We hypothesized that Ronan's beat-keeping performance, and her response to phase and tempo perturbations, would be well fit by simple models of phase and period coupling.

## Methods

### Subject

The subject was “Ronan,” a 7-year-old female California sea lion (NOA0006602), who was housed at Long Marine Laboratory at the University of California Santa Cruz. Ronan was a healthy individual that was placed into captivity around age one after repeated stranding incidents and rescues. She previously participated in a study examining her ability to synchronize to auditory rhythms (Cook et al., 2013). In brief, Ronan was trained to match regular head movement to a simple isochronous stimulus at tempos of 80 and 120 beats per minute (bpm). She then successfully generalized the behavior to novel tempos of 72, 88, 96, 108, and 132 bpm with the simple stimulus, and to novel musical stimuli at tempos of 104, 108, 117, 124, 130, 137, and 143 bpm. Following data collection for Cook et al. (2013), Ronan received intermittent “practice” sessions (typically no more than one per week) with familiar simple stimuli and several novel musical stimuli. During this time, Ronan also participated in several other cognitive and perceptual studies unrelated to rhythm (Reichmuth et al., 2013; Cunningham et al., 2014a,b; Cook et al., 2015; Cunningham and Reichmuth, 2016).

The current experiment occurred from September 2015 to January 2016. During this time, Ronan received a daily diet of 5.7–6.6 kg of freshly thawed, cut herring and capelin fish. She was maintained at a healthy weight of ~72 kg, and her diet was not constrained for experimental purposes. Ronan typically participated in five sessions per week, receiving ~40% of her diet during these experimental sessions.

The study was conducted without harm under National Marine Fisheries Service marine mammal research permits 14535 and 18902, with the approval and oversight of the Institutional Animal Care and Use Committee at the University of California Santa Cruz.

### Apparatus

Testing occurred in a 3.6 × 5.2 m enclosure containing a 1.2 m deep, 2.25 m square pool, and surrounding deck space. The experimental setup (similar to that used in Cook et al., 2013) consisted of a 1.1 × 1.5 m painted wooden panel mounted vertically in the doorway of the enclosure. A 0.8 × 0.3 m raised wooden platform was placed on the deck facing the panel, 0.4 m away. Ronan used this platform to find and maintain a consistent stationing position prior to each trial. She rested her foreflippers on the platform while directly facing the panel, and could then move her head freely without touching the panel. An assistant sitting quietly outside the enclosure and behind the panel delivered fish rewards through a short length of PVC pipe mounted in the panel. The experimenter observed Ronan's real-time performance from behind the panel through a 9 cm diameter convex mirror placed 2 m to the side of the flipper station. Both the experimenter and the assistant were concealed from Ronan's view during all trials.

Each session was recorded on a GoPro Hero 2 camera mounted inside the enclosure, 0.25 m above the convex mirror. The auditory stimuli were projected from an Advent AV570 amplified speaker placed ~1 m from Ronan and from the camera. The absolute broadband received level of the brief auditory stimuli presented through the speaker was ~100 dB_peak_ (re 20 μPa); the equivalent sensation level was 60–80 dB at the frequency of the test stimuli based on species-typical hearing sensitivity (Reichmuth et al., 2013). The level of the stimulus was established to ensure saliency of the auditory cues in an outdoor, coastal environment.

### Stimuli

Stimuli were repetitive click tracks created in Audacity, an open-source audio editing program. The clicks comprised two overlaid pure tones of 659 and 1319 Hz for a duration of 10 ms, as was used in Cook et al. (2013). Each track began with a series of beats at a steady rate followed by a single perturbation of either phase or tempo at a magnitude of ±25, ±15, ±8, or ±3% of the inter-onset interval (IOI, equivalent to 60 divided by the tempo in beats per minute). For example, a +15% shift of the 85 bpm condition would be 73.913 bpm. The perturbations were introduced at a different beat for each condition (between 16 and 25 beats after the beginning of the trial) to prevent prediction of the onset location. Primary testing was completed at a base tempo of 85 bpm (705.88 ms IOI), to which Ronan had not previously been exposed; Table 1 lists the perturbation tempos, and their corresponding IOI values. We also tested Ronan with stimulus perturbations at two additional novel base tempos (94.444 and 77.273 bpm, ±10% of the 85 bpm condition, see Supplementary Materials) independently of each other and the main tempo.

**Table 1:** **Tempo and inter-onset interval perturbation values referenced to the baseline (no perturbation) condition**.

	**Tempo (bpm)**	**IOI (ms)**
Baseline	85.000	705.882
+25%	68.000	882.353
+15%	73.913	811.765
+8%	78.704	762.353
+3%	82.524	727.059
−3%	87.629	684.706
−8%	92.391	649.412
−15%	100.000	600.000
−25%	113.333	529.412

### General procedure

The auditory stimulus was started after Ronan calmly positioned at the flipper station and oriented toward the panel. The trial was ended after a predetermined performance criterion of 20 or 40 consecutive, apparently entrained bobs (termed “good” bobs) as judged by the experimenter in real time, similar to the procedure used in Cook et al. (2013). Transfer trials (i.e., trials when a perturbation was presented) were run to a criterion of 20 good bobs following the perturbation, and baseline trials (those with no perturbation) were run to a criterion of 40 good bobs starting at the beginning of the trial. At the beginning of each session, two “warm-up” trials (at the base tempo with no perturbation) were presented to confirm stimulus control of the behavior and run to a criterion of 15–30 apparently entrained beats. Each trial was terminated with a previously conditioned reinforcer (a sharp whistle blown by the experimenter that marked the last bob in the criterial run) followed by a reward of two whole capelin fish offered to Ronan through the feeding port in the panel. Ronan then entered the pool for a small fish reward and returned to the flipper station to begin the next trial.

One experimental replicate at a given base tempo encompassed 24 trials: one trial at each test condition (eight phase changes and eight tempo changes for a total of 16 perturbations) and eight unperturbed trials at the base rate. One session, equivalent to one half of a replicate series, consisted of two warm-up trials at the base rate followed by a randomly-shuffled sequence consisting of four baseline (unperturbed) trials, four tempo perturbations, and four phase perturbations. The perturbations for both phase and tempo were each counterbalanced to ensure an equal number of positive and negative shifts per session. Sessions were broken into three blocks of four stimulus presentations each (not including the two warm-up trials), with a short 30 s break between each block, in which Ronan received four to five half capelin while swimming calmly in the water.

Ronan completed 10 replicates of the 85 bpm test series during 20 sessions; that is, she completed 10 trials with each of the 16 phase and tempo perturbations (*n* = 160 perturbation trials, *n* = 80 baseline trials). Subsequently, she completed a single replicate with the two additional base tempos over a total of four sessions (*n* = 32 perturbation trials, *n* = 16 baseline trials).

During two sessions, trials were interrupted by external factors before the perturbation occurred: once by vocalizations from an animal in a neighboring enclosure, and once by beeping from a truck in close proximity to the testing facility. In these cases, the experimenter immediately stopped the interrupted trial and proceeded as though the trial had been completed. The aborted trial was retested at the end of the session, and the interrupted trials were not included in any analysis.

### Video analysis

We recorded each session at a frame rate of 120 frames per second (equivalent to a resolution of 8.333 ms per frame). Although Ronan's behavior was continuous, the video data is necessarily binned into windows of 8.333 ms, which introduces some small margin of error into any analysis of precise timing. Nevertheless, a single frame represents, at most, 2% of the IOI.

The primary measure of Ronan's performance was selected as the coincidence of the nadir of her head position with the onset of an auditory beat. Specifically, the height of the tip of the nose was used as the marker for the inflection point. An observer using frame-by-frame analysis determined the time of the lowest point for each head bob, with trials viewed in AvsPmod, an open-source video editing program. When more than one frame appeared to show the lowest point, the first of these frames was selected.

Video footage was analyzed independently by two observers. Inter-observer reliability was determined using a common subset of practice trials. Out of 136 bobs, the observers agreed on the frame number corresponding to the lowest point on 127 of them, and the nine disagreements fell within one frame of each other; this means that 100% of the observations were within 8.333 ms. Considering the 10,777 frames over these 136 bobs, the calculated Cohen's kappa (0.933) indicated very high inter-observer agreement.

To compare the observed head movements to the timing of the auditory stimuli, we combined the movement data from video analysis with the timing of beat onset determined from the corresponding camera audio, sampled at 48 kHz. For each trial, we located the onset of the first beat by visual analysis of the waveform to the nearest millisecond using Audacity, and then calculated times of subsequent beats based on the stimulus tempo and perturbation location. Because the speaker, the camera, and Ronan were approximately equidistant, we considered the sound travel time from the speaker to Ronan as approximately equal to the sound travel time from the speaker to the camera. Therefore, we used the audio from the camera for timing and did not consider the sound travel time in any subsequent calculations.

### Statistical analysis

We quantified Ronan's performance with circular statistics. As the analysis was focused on performance following perturbation rather than overall statistical similarity between movement and beat, analysis of each transfer trial was restricted to a subset of bobs: specifically, the 10 bobs preceding the perturbation and the 20 bobs following. In baseline trials, where there was no perturbation, the entire trial was included.

In several trials (*n* = 10), Ronan exhibited “double bobs” where she bobbed twice for a given beat. This typically occurred during the +25% tempo condition of the 85 bpm base tempo, when she continued moving at the original rate such that when the first shifted beat occurred, her head was near the highest point of the bob rather than the lowest. In response to this beat, Ronan immediately nodded her head in a smaller bobbing motion before slowing her overall movement to adapt to the new tempo. These outlier bobs were easily identified by the shift in angle of her head in relation to her neck, and were excluded from the analysis. An example of a double bob can be seen in the first trial of Supplementary Video 1.

First, the relative phase angle between each head bob and the nearest stimulus beat was calculated using Equation (2).

(2) $$\begin{array}{l} \phi_{n}=2\pi \left(\frac{t_{bob_{n}}-t_{beat_{n}}}{I_{n}}\right) \end{array}$$

The phase is expressed, in radians, as the time between a head bob (*t*_*bob*_*n*__) and the nearest stimulus beat (*t*_*beat*_*n*__), as a proportion of the inter-onset interval of the beats surrounding the head bob (*I*_*n*_). For all bobs, the relative phase angle was inherently restricted to a range of −π to π radians, as no bob occurred more than π radians away from its nearest beat.

We calculated the mean relative phase angle ($\bar{\phi }$) and mean vector length (*r*) for each trial using the argument (Equation 3) and modulus (Equation 4), respectively, of the sums of the angles as complex numbers.

(3) $$\begin{array}{rcl} \bar{\phi } & = & \frac{1}{n}\text{arg}\overset{n}{\underset{j=1}{\sum }}e^{i\cdot \phi_{j}} \end{array}$$

(4) $$\begin{array}{rcl} r & = & \frac{1}{n}\text{abs}\overset{n}{\underset{j=1}{\sum }}e^{i\cdot \phi_{j}} \end{array}$$

The mean vector length, which indicates the concentration of the mean angle, ranges from 0 (no mean angle) to 1 (perfect concordance of angles). Together, the mean relative phase angle and mean vector length specify the strength of Ronan's performance on a given trial. For each trial, we used the *V*-test to determine whether Ronan's performance was significantly different from a mean relative phase of 0, which would indicate perfect synchrony with the stimulus (Zar, 1999). Again, we included both pre-perturbation (10 preceding) bobs and post-perturbation (20 following) bobs in this analysis to provide an evaluation of her synchronization across each transfer trial; all bobs within each baseline trial were included.

Relative phase angles for each beat were also averaged across replicates to obtain an average trial for the baseline and each perturbation type. We used these averaged trials to fit the oscillator models.

### Model fitting

The nonlinear equations that describe rhythmic behavior are often explained using a “circle map,” an equation that produces a set of phases which predict the phase of a stimulus event relative to the onset of the behavioral oscillation (Pikovsky et al., 2001; Large and Palmer, 2002).

(5) $$\begin{array}{l} \phi_{n+1}=\phi_{n}+2\pi f_{ronan}\left(t_{n+1}-t_{n}\right)-\alpha \text{ sin }\phi_{n}\;\left({mod}_{-\pi ,\pi }2\pi \right) \end{array}$$

Equation (5) states that the phase of each successive auditory event (ϕ_*n*+1_)—in this case, the onset of the click stimulus—is determined by the current auditory event's relative phase (ϕ_*n*_), the frequency of the stimulus relative to the oscillator's frequency (expressed as the product of the current period of the stimulus (*t*_*n*+1_−*t*_*n*_) and the current radian frequency of the oscillator (ω_*n*_ = 2π*f*_*ronan*_), and a stimulus coupling (i.e., sine of the current auditory event's relative phase modulated by a coupling factor, α). The coupling factor indicates how strongly the relative phase of the oscillator is affected by the stimulus. Because phase is a circular value, the resulting phase is taken modulo 2π (the remainder after dividing the phase by 2π) and normalized to the range of −π to π.

If Ronan were to bob her head at exactly the same rate as the stimulus, the number of bobs to correct for being ahead of or behind the beat would depend solely on the phase coupling factor, α: a high coupling factor would mean a very quick adaptation to the stimulus, while a low coupling factor would mean a slower adaptation. The optimal value for the phase coupling factor is 1.0, which is the largest value that does not result in overcorrection. A value >2.0 causes the equation to become unstable (Pikovsky et al., 2001).

If, on the other hand, Ronan's period were different from the stimulus period, she still might be able to adapt to a steady phase, but it would be at a non-zero phase. This non-zero phase can be calculated with Equation (5) by assuming that ϕ_*n*+1_ = ϕ_*n*_ and solving for ϕ_*n*_. However, if the phase coupling factor (α) was not sufficiently large, she might never adapt to a steady phase and instead would start to “phase-wrap.” Therefore, phase adaptation alone does not guarantee perfect synchronization; period adaptation is required as well. This is described by a supplemental equation to the circle map.

(6) $$\begin{array}{l} \omega_{n+1}=\omega_{n}-\beta \text{ sin }\phi_{n} \end{array}$$

From Equation (6), we can see that each successive oscillator radian frequency (ω_*n*+1_)—in this case, the oscillator radian frequency is 2π multiplied by the inverse of the time between two successive head bobs—is dependent on the oscillator radian frequency of the current beat (ω_*n*_) and a different stimulus coupling (the sine of the relative phase of the current beat, modulated by a different coupling factor, β). Again, this coupling factor indicates how strongly the oscillator period is affected by the stimulus.

Thus, in order to completely synchronize, an oscillator needs to adapt both phase and period. Together, Equations (5) and (6) accurately model not only the entrainment of human performers to a simple repeating stimulus, but to stimuli with multiple changes in phase and tempo over the course of the stimulus.

We fit a deterministic version of Equations (5) and (6) to the circularly averaged trial from each condition. α and β were varied to minimize the root mean squared error (RMSE) of the test model compared to Ronan's data. The range used for fitting α was 0.4–2.0 in increments of 0.1, and the range used for fitting β was 0.01–0.70 in increments of 0.01. Because Equation (5) describes the phase of the stimulus relative to the oscillator, the phases produced by the model are opposite of the measured behavioral data, which indicates the phase of the oscillator to the stimulus. In other words, ϕ_*model*_ = −ϕ_*measured*_.

#### Parameter regression

To identify any potential relationship between either of the coupling parameters and the perturbation magnitude, we regressed both α and β as a function of condition against perturbation magnitude.

## Results

**Figure 1:**
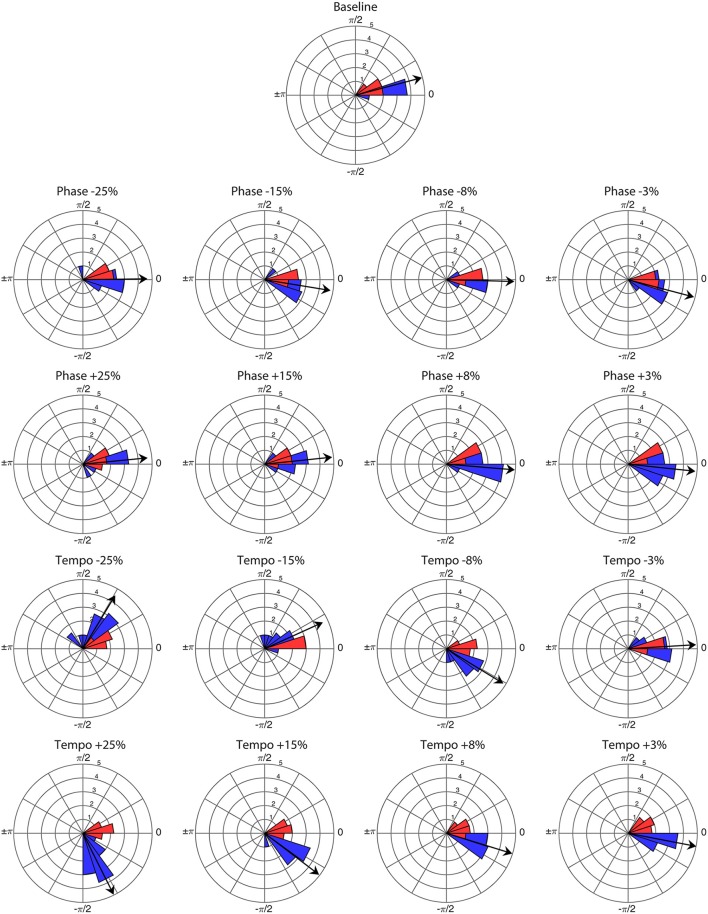
**Angular distribution of phase angles for the first presentation of each perturbation condition (***n*** = 16) and baseline trial (***n*** = 1) at a base tempo of 85 bpm**. Red wedges show 10 bobs prior to the perturbation and blue wedges show 25 bobs following. The radius of each wedge represents the square root of the number of bobs in that wedge, so that the area of each wedge is equal to the number of bobs. The arrow indicates the mean vector of the post-perturbation bobs, with length normalized to the outer radius of the plot. Note that angles >0 represent bobs trailing the stimulus beat, and angles <0 represent bobs leading the stimulus beat.

**Figure 2:**
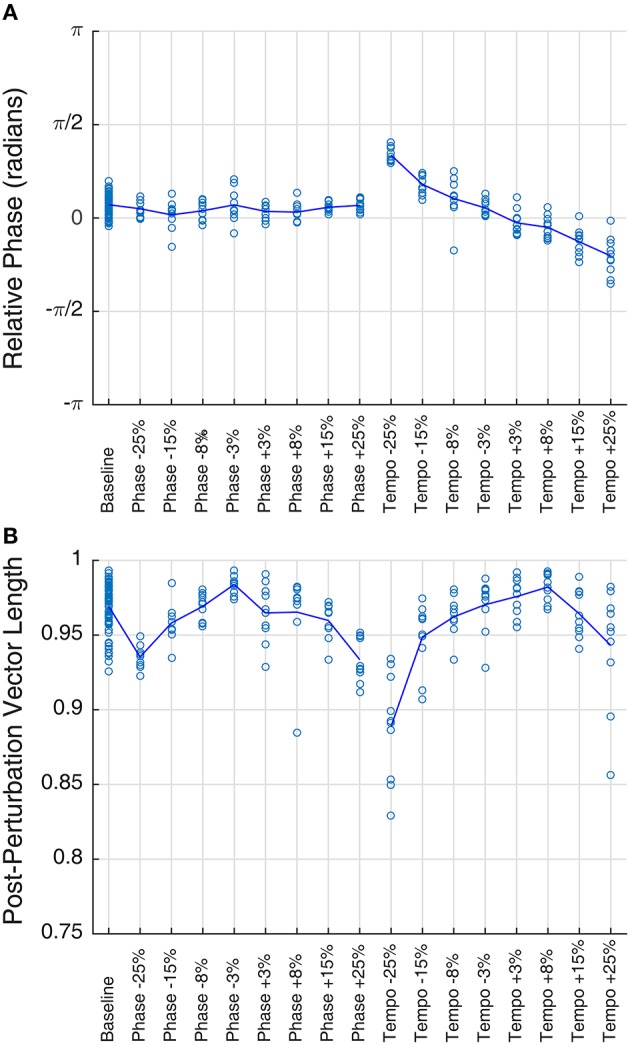
**Mean phase (A) and vector length (B) of Ronan's post-perturbation bobs at a base tempo of 85 bpm, grouped by condition**. Each trial is plotted as a circle, and the mean for each condition is represented by the line. Mean phase shows a linear trend with tempo changes (right portion of plot **A**), a trend described in a prior study of Ronan's rhythmic entrainment ability (Cook et al., 2013). In all cases, mean vector length falls between 0.88 and 0.99, indicating a very high concordance of phases within the trial.

Ronan successfully entrained to all stimuli and perturbations at the base tempo of 85 bpm (see Supplementary Video 1). For all trials, the critical value of the *V*-test was >3.4, indicating that the distribution of bobs was significantly nonrandom (*p* < 0.001) with respect to 0 radians. Thus, Ronan's head movements were strongly correlated with the beat on both baseline trials and transfer trials containing a perturbation event. Figure 1 displays the correspondence of the angular distributions of the initial presentations of each condition, and Figure 2 displays the similarity between performances in all trials. Her subsequent performance on the two additional base tempos (94.444 and 77.273 bpm) showed similar results: she successfully entrained to all stimuli and perturbations (see Supplementary Tables 1, 2 and Supplementary Figures 1–3).

Ronan's performance on baseline and transfer trials revealed rapid entrainment to the base tempo, with performance stabilizing within the first four beats (Supplementary Video 1), as previously observed by Cook et al. (2013). Unexpectedly, her performance with all tempos and all stimuli showed a slight phase progression over the course of each trial: the average slope of relative phase per beat across all trials was −0.0206, and the slope was different from zero for the majority of trials (*n* = 80 baseline trials, *n* = 62 phase perturbation trials, *n* = 55 tempo perturbation trials, *p* < 0.05). This represents a deviation from her previous performance (Cook et al., 2013). However, this deviation was consistent across replicates and conditions.

**Figure 3:**
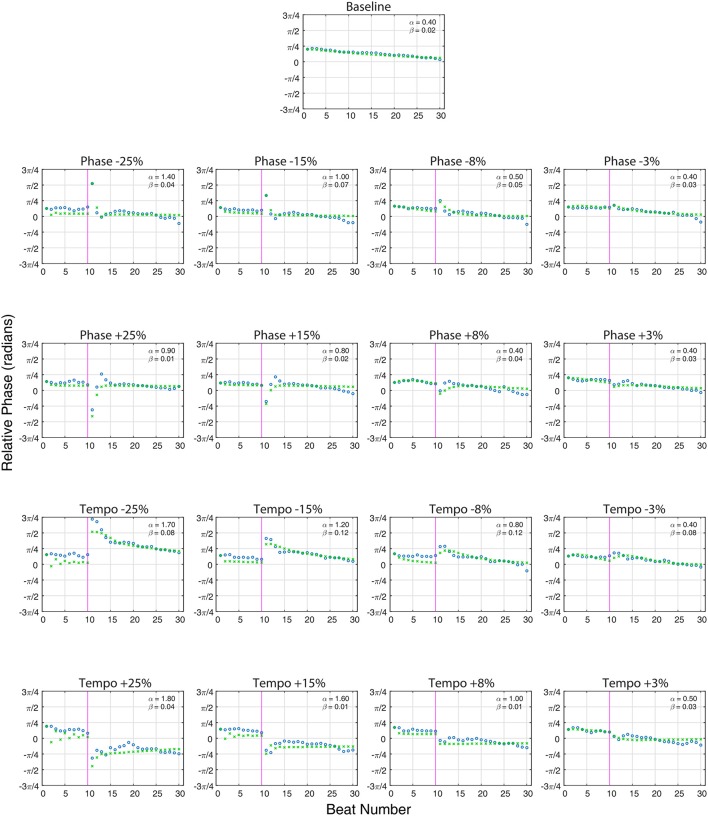
**The averaged relative phase in radians (Y-axis) over successive beats (X-axis) for averaged baseline trials (***n*** = 80, top) and each averaged perturbation condition (***n*** = 10 per condition) at a base tempo of 85 bpm**. Ronan's results (circles) are plotted against the model predictions (x's). The close alignment of the model predictions to the experimentally obtained values indicate that Ronan's responses to these perturbations are consistent with the model of coupled oscillation. On perturbation conditions, the vertical line at 10 beats indicates the onset of the tempo or phase shift indicated at the top of the plot. Phase (α) and period (β) coupling factors are noted in the upper right portion of each plot.

**Figure 4:**
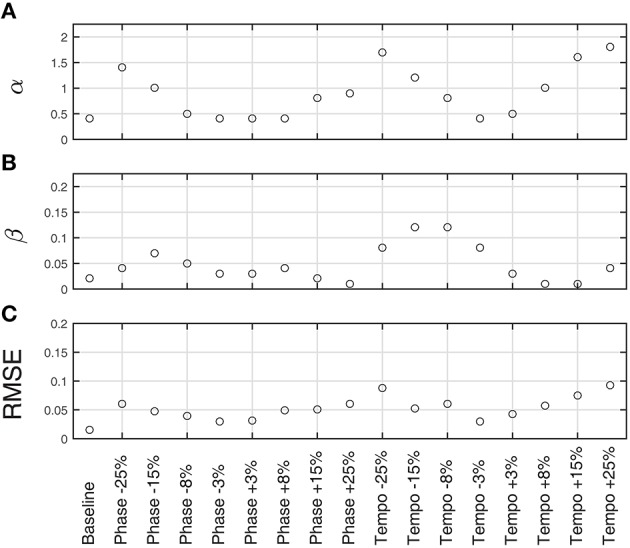
**Fitted model parameters and RMSE for averaged trials for each condition at 85 bpm (***n*** = 80 baseline trials, ***n*** = 10 perturbation trials per condition)**. α **(A)** represents the strength of phase coupling, or how strongly the stimulus affects the phase of the model's behavior. β **(B)** represents the strength of the period coupling. On average, the magnitude of phase coupling was 20 times stronger than the magnitude of the period coupling, suggesting that Ronan is primarily using phase adaptation to match the stimulus. Also of note is that α is strongly affected by perturbation magnitude. RMSE **(C)** is the root mean squared error of the fitted model compared to Ronan's behavior.

Figure 3 shows the model fit compared to Ronan's pooled performance for each condition. Table 2 and Figure 4 show the fitted coupling parameter values and final RMSE for each condition. RMSE was very low for all conditions, with an average value of 0.0518 radians. Phase coupling was strong; across all conditions, the average parameter value was 0.894. Loehr et al. (2011) found that human subjects performing a comparable task (playing a piano keyboard to a metronome with a changing tempo) had an average phase coupling parameter value of 0.875, quite close to Ronan's. Ronan's observed period coupling was much weaker, averaging 0.0471 across all conditions. This is quite low compared to the subjects in the Loehr study, who had an average period coupling parameter value of 0.450.

**Table 2:** **Phase (α) and Period (β) coupling parameter values and Root Mean Squared Error (RMSE) for model fits of Ronan's experimental data at 85 bpm**.

Condition	α	β	RMSE
Baseline	0.4	0.02	0.0153
Phase −25%	1.4	0.04	0.0599
Phase −15%	1.0	0.07	0.0473
Phase −8%	0.5	0.05	0.0402
Phase −3%	0.4	0.03	0.0306
Phase +3%	0.4	0.03	0.0307
Phase +8%	0.4	0.04	0.0484
Phase +15%	0.8	0.02	0.0515
Phase +25%	0.9	0.01	0.0601
Tempo −25%	1.7	0.08	0.0871
Tempo −15%	1.2	0.12	0.0516
Tempo −8%	0.8	0.12	0.0602
Tempo −3%	0.4	0.08	0.0305
Tempo +3%	0.5	0.03	0.0433
Tempo +8%	1.0	0.01	0.0571
Tempo +15%	1.6	0.01	0.0744
Tempo +25%	1.8	0.04	0.0921

We also observed a significant positive linear relationship between phase coupling magnitude and absolute perturbation magnitude [RMSE = 0.2800, *F*_(1, 15)_ = 35.1, *p* < 0.0001]. Period coupling, on the other hand, showed a significant negative linear relationship with perturbation magnitude [RMSE = 0.0288, *F*_(1, 15)_ = 9.02, *p* < 0.01].

## Discussion

Ronan's performance with novel tempos containing embedded tempo and phase perturbations showed remarkable ability to adapt quickly and accurately to synchronize her body motion to the temporal features of the auditory stimulus stream. Moreover, her beat keeping (ranging from 61.818 to 125.925 bpm across the three base tempos) and adaptation (±25% of the IOI) impressively fit models of co-oscillation, drawn from physics and validated in human beat-keeping experiments. The findings show a strong similarity between dynamics of Ronan's performance and human performance, and parsimony suggests these are rooted in similar and conserved neural mechanisms rather than species-specific adaptations. However, these results by themselves are not dispositive, and more comparative data are needed to fully resolve the debate over underlying mechanisms.

Through the lens of neural resonance, we see that Ronan's beat-keeping behavior in response to stimulus perturbations compared strongly to that measured in humans in four ways: (1) flexibility in tempo matching was evident in her behavior throughout testing, (2) changes in phase and tempo were matched through both phase and period adaptation, (3) phase adaptation was stronger than tempo adaptation, and (4) reduced sensitivity to smaller perturbations was observed (discussed below).

Ronan's performance in this study differed from human performance in two important ways, related to (1) phase coupling, and (2) period adaptation. For most perturbation trials, phase coupling (α) varied based on perturbation magnitude, dramatically increasing for larger magnitude shifts (perturbations >8%). The significant linear relationship between α and absolute perturbation magnitude suggests that the more noticeable alterations induced a larger change in coupling. In most human studies, phase coupling has been considered more or less constant (for review see Repp, 2005; Large, 2008), so Ronan's variable coupling strength is a novel discovery. However, her performance does align with findings in humans that larger perturbations are more noticeable because they represent more significant violations of expectation of where the next beat should occur (Large and Jones, 1999). It also suggests another similarity to humans: the just-noticeable difference in humans for tempo changes of a single interval (equivalent to a change in phase) is ~6% (Drake and Botte, 1993). Ronan's results here imply that she did not readily perceive the ±3 or ±8% phase perturbations or the ±3% tempo perturbations, similar to what might be expected in human performance based on available research.

The second divergence in Ronan's behavior relative to that of humans is decreased period adaptation. While human studies have shown typical period coupling values between 0.3 and 0.8 (Loehr et al., 2011), Ronan's period coupling values did not exceed 0.2. Again, it is important to note that the human comparison is imperfect. Human subjects played a melody on the keyboard with a metronome, as opposed to a single discrete repeating movement. Furthermore, rather than a single sudden shift, the tempo changed continuously following the shift onset.

Additionally, Ronan had a larger phase/tempo offset than typically seen in humans (e.g., Repp, 2005; Repp and Su, 2013): her starting relative phase showed a direct linear correlation with IOI, with faster tempos effecting a starting phase further behind the beat, a trend described previously in Cook et al. (2013).

These differences relative to human subjects may be rooted in behavioral aspects of Ronan's performance. The gradual phase progression on all trials and changing phase coupling strength for larger perturbations suggest that Ronan used a specific strategy to entrain to these stimuli. Although she showed reliable phase and tempo matching throughout the experiment, her precision dramatically increased following relatively large perturbations. One possible interpretation is that basic beat keeping with simple metronomic stimuli is quite easy for Ronan following her extensive training with these and more complicated stimuli. Perhaps she uses a motor heuristic to produce “good enough” entrainment without employing any significant attentive effort. However, following a perturbation, realigning her movement with the beat may require greater attention. This could then drive an up-regulation of auditory motor networks, leading to increased coupling and greater performance. There is extensive evidence that human beat-keeping performance is heavily reliant on intent and attention (see Large and Jones, 1999; Repp, 2005). Furthermore, “task-positive” networks in humans—attention-driven brain networks that up-regulate in-network functional connectivity (i.e., co-synchrony across nodes) during rigorous mental action—include motor and motor planning regions (Fox et al., 2005; Bardouille and Boe, 2012). Increased attention to the stimulus following perturbation could therefore lead to increased resonance between the neural oscillators of interest.

In most respects, Ronan's beat-keeping performance was as precise and reliable as that observed in human studies, and was well fit by models of coupled oscillation. Ronan's obvious ability to adaptively entrain her body movements to auditory rhythms extends the findings reported for this subject by Cook et al. (2013). Although the current experiment did not explicitly test adaptation to phase or tempo change in more complex stimuli, Ronan has successfully entrained to human-generated music that contains natural variability in both phase and tempo (Cook et al., 2013). Not only does this support the likelihood of shared mechanisms, it emphasizes Ronan's usefulness as a comparative model to study other aspects of rhythmic entrainment. In addition, Ronan's beat keeping did not emerge *de novo*—she received explicit and extensive operant (positive reinforcement) training. Therefore, she may serve as a model for training other non-human beat keepers. Supplemental testing with Ronan and with additional non-human subjects should clarify the mechanisms supporting beat-keeping ability and resolve whether these mechanisms are evolutionarily conserved. Further exploration of these results may also improve understanding of other facets of human musical ability. Resonance of neural oscillators with an external stimulus has been proposed as the foundation for many areas of music perception and cognition, including pitch and meter perception (see Large, 2008 for a review).

Patterns of neural oscillations have been observed in every nervous system examined (Glass, 2001). The basic physics of the structure of neural oscillators shows that if stimulated rhythmically, they will synchronize. The finding that non-human as well as human beat keeping is consistent with models of neural resonance supports a parsimonious explanation of beat-keeping behavior as arising from basic principles of nervous system behavior. That being said, the tendency of linked neural populations to co-oscillate could be only the beginning of an understanding of sensorimotor synchronization. While coupled oscillation between neural populations may be necessary and sufficient for supporting a general faculty for beat keeping, great potential still exists for variability in the dynamics of beat-keeping behavior. First and foremost, animals may differ in connection strengths between relevant neural populations. This could be due to differences in anatomical connectivity, or differences in functional connectivity in these brain circuits, which can change with learning, across development, and dynamically with attention, intention, and other psychological factors. To date, the field of comparative rhythm has focused on answering the question “which species can keep a beat?” If basic and conserved neural mechanisms support entrainment intrinsically, the more productive question is this: “How can we use sensorimotor synchronization paradigms as a comparative tool to better understand brain function and behavior across species and contexts?”

## Author contributions

AR, PC, and CR designed the study; AR conducted all experiments; and AR, PC, CR, and EL analyzed the data, interpreted the results, and wrote the manuscript.

## Funding

Support for this work was provided in part by the Special Projects Fund of the Pinniped Cognition and Sensory Systems Laboratory, and a grant to CR from the International Association of Oil and Gas Producers through the Exploration and Production (E&P) Sound and Marine Life Joint Industry Programme (Award 22-07-23).

### Conflict of interest statement

The authors declare that the research was conducted in the absence of any commercial or financial relationships that could be construed as a potential conflict of interest.
