# Supplementary material for: Beat Keeping in a Sea Lion As Coupled Oscillation: Implications for Comparative Understanding of Human Rhythm
Source: Front Neurosci. 2016 Jun 3;10:257. doi: 10.3389/fnins.2016.00257 (PMC4891632; doi:10.3389/fnins.2016.00257)
Supplement: Supplementary file 1 [file Table1.DOCX]

Supplementary Material

Beat Keeping in a Sea Lion as Coupled Oscillation: Implications for Comparative Understanding of Human Rhythm

Andrew A. Rouse*, Peter F. Cook, Edward W. Large, Colleen Reichmuth

*** Correspondence:** Corresponding Author: arouse@ucsc.edu

# Supplementary Figures and Tables

Supplementary Table 1. Tempo and Inter-Onset Interval Perturbation Values Referenced to the Baseline (No Perturbation) Condition for Base Tempos of 94.444 bpm and 77.273 bpm

|  | 94.444 bpm | | 77.273 bpm | |
| --- | --- | --- | --- | --- |
|  | Tempo (bpm) | IOI (ms) | Tempo (bpm) | IOI (ms) |
| Baseline | 94.444 | 635.297 | 77.273 | 776.468 |
| +25% | 75.555 | 794.121 | 61.818 | 970.585 |
| +15% | 82.125 | 730.592 | 67.194 | 892.938 |
| +8% | 87.448 | 686.121 | 71.549 | 838.585 |
| +3% | 91.693 | 654.356 | 75.022 | 799.762 |
| -3% | 97.365 | 616.238 | 79.663 | 753.174 |
| -8% | 102.657 | 584.473 | 83.992 | 714.35 |
| -15% | 111.111 | 540.003 | 90.909 | 659.998 |
| -25% | 125.925 | 476.473 | 103.031 | 582.351 |
