# Supplementary material for: Beat Keeping in a Sea Lion As Coupled Oscillation: Implications for Comparative Understanding of Human Rhythm
Source: Front Neurosci. 2016 Jun 3;10:257. doi: 10.3389/fnins.2016.00257 (PMC4891632; doi:10.3389/fnins.2016.00257)
Supplement: Supplementary file 2 [file Table2.DOCX]

Supplementary Material

Beat Keeping in a Sea Lion as Coupled Oscillation: Implications for Comparative Understanding of Human Rhythm

Andrew A. Rouse*, Peter F. Cook, Edward W. Large, Colleen Reichmuth

*** Correspondence:** Corresponding Author: arouse@ucsc.edu

# Supplementary Figures and Tables

Supplementary Table 2. Phase (α) and Period (β) Coupling Parameter Values and Root Mean Squared Error (RMSE) for Model Fits of Ronan’s Experimental Data at 94.444 bpm and 77.273 bpm

|  | 94.444 bpm | | | 77.273 bpm | | | |
| --- | --- | --- | --- | --- | --- | --- | --- |
| Condition | α | β | RMSE | α | β | RMSE |  |
| Baseline | 0.5 | 0.01 | 0.0186 | 1.4 | 0.70 | 0.0248 |  |
| Phase -25% | 1.0 | 0.01 | 0.1018 | 1.9 | 0.70 | 0.0645 |  |
| Phase -15% | 0.6 | 0.01 | 0.0748 | 1.4 | 0.30 | 0.1598 |  |
| Phase -8% | 0.5 | 0.01 | 0.0393 | 1.0 | 0.21 | 0.0869 |  |
| Phase -3% | 0.5 | 0.01 | 0.0435 | 1.0 | 0.70 | 0.1056 |  |
| Phase +3% | 0.5 | 0.01 | 0.0457 | 0.8 | 0.22 | 0.0689 |  |
| Phase +8% | 0.4 | 0.01 | 0.0563 | 1.5 | 0.70 | 0.0503 |  |
| Phase +15% | 0.6 | 0.01 | 0.0596 | 1.6 | 0.70 | 0.0617 |  |
| Phase +25% | 0.8 | 0.01 | 0.0535 | 0.8 | 0.20 | 0.0911 |  |
| Tempo -25% | 1.7 | 0.03 | 0.0957 | 2.0 | 0.14 | 0.0581 |  |
| Tempo -15% | 1.3 | 0.11 | 0.0710 | 1.3 | 0.36 | 0.0291 |  |
| Tempo -8% | 0.8 | 0.15 | 0.0413 | 1.1 | 0.33 | 0.0464 |  |
| Tempo -3% | 0.5 | 0.06 | 0.0494 | 0.8 | 0.33 | 0.0678 |  |
| Tempo +3% | 0.8 | 0.01 | 0.0444 | 0.9 | 0.10 | 0.1425 |  |
| Tempo +8% | 1.3 | 0.01 | 0.0427 | 0.8 | 0.01 | 0.0936 |  |
| Tempo +15% | 1.7 | 0.16 | 0.0726 | 1.5 | 0.01 | 0.0579 |  |
| Tempo +25% | 2.0 | 0.01 | 0.1279 | 1.7 | 0.01 | 0.0572 |  |
