# Supplementary figures and images for: Beat Keeping in a Sea Lion As Coupled Oscillation: Implications for Comparative Understanding of Human Rhythm
Source: Front Neurosci. 2016 Jun 3;10:257. doi: 10.3389/fnins.2016.00257 (PMC4891632; doi:10.3389/fnins.2016.00257)

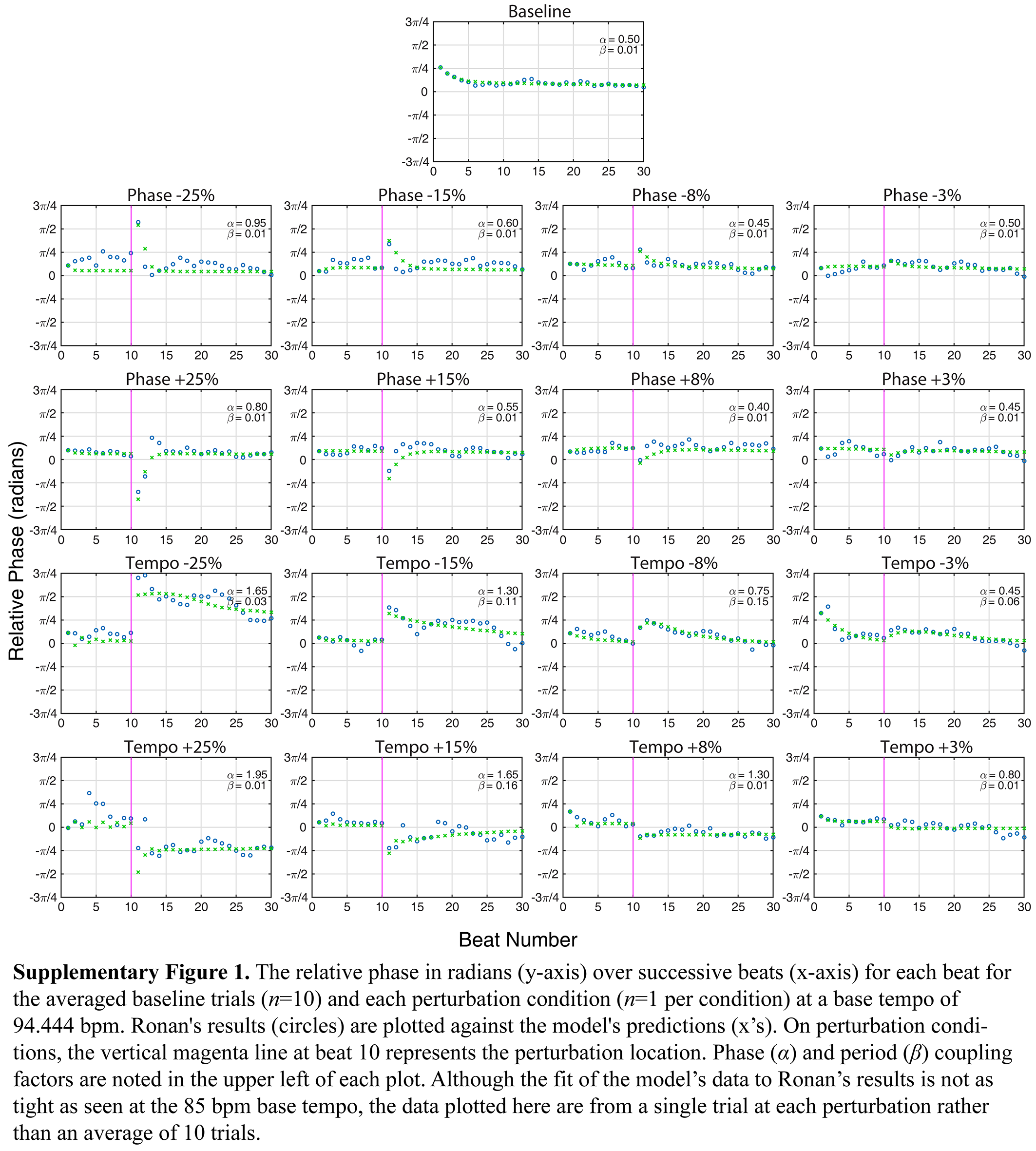

Supplement: Supplementary file 4 [file Image1.tif]

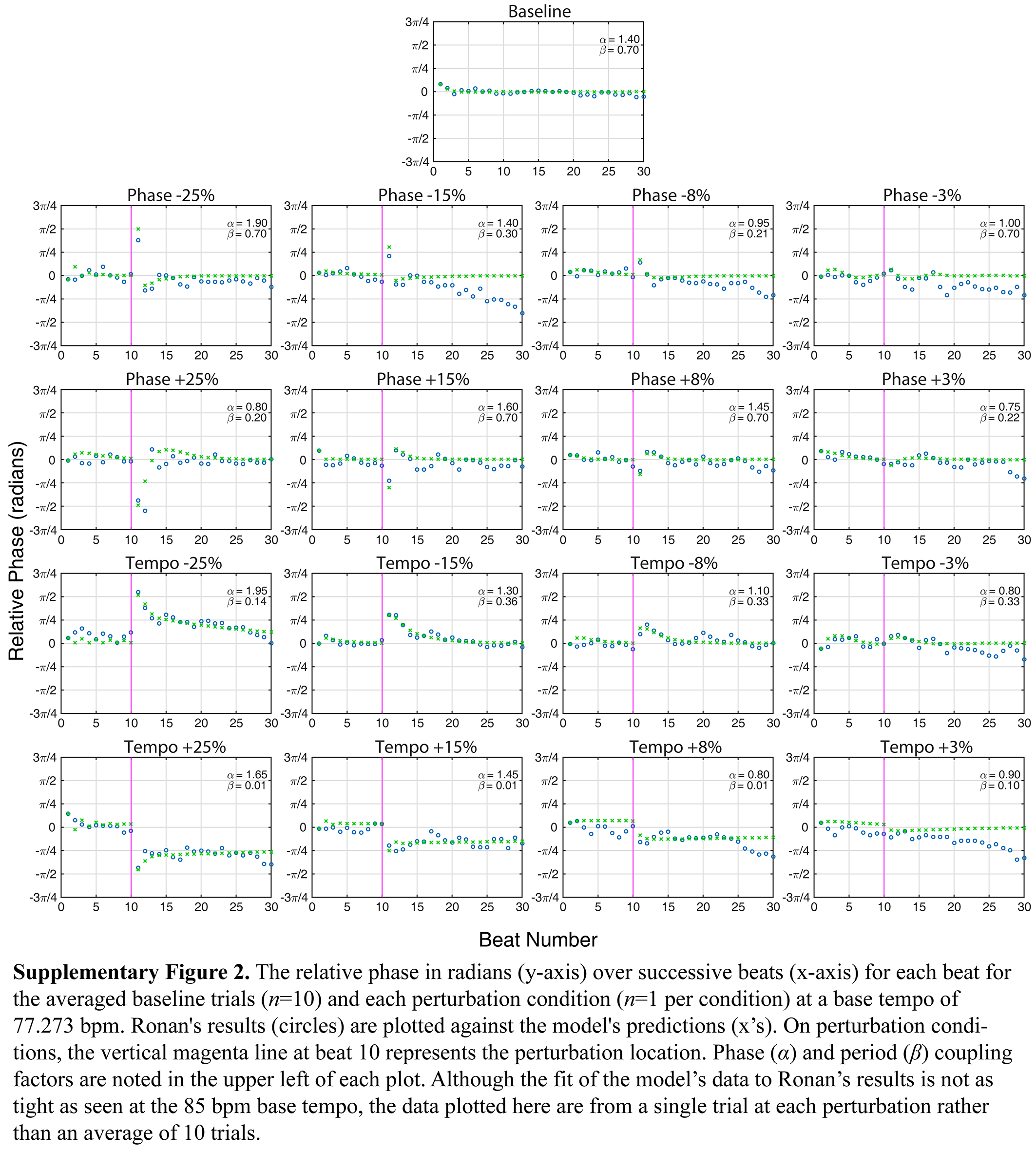

Supplement: Supplementary file 5 [file Image2.tif]

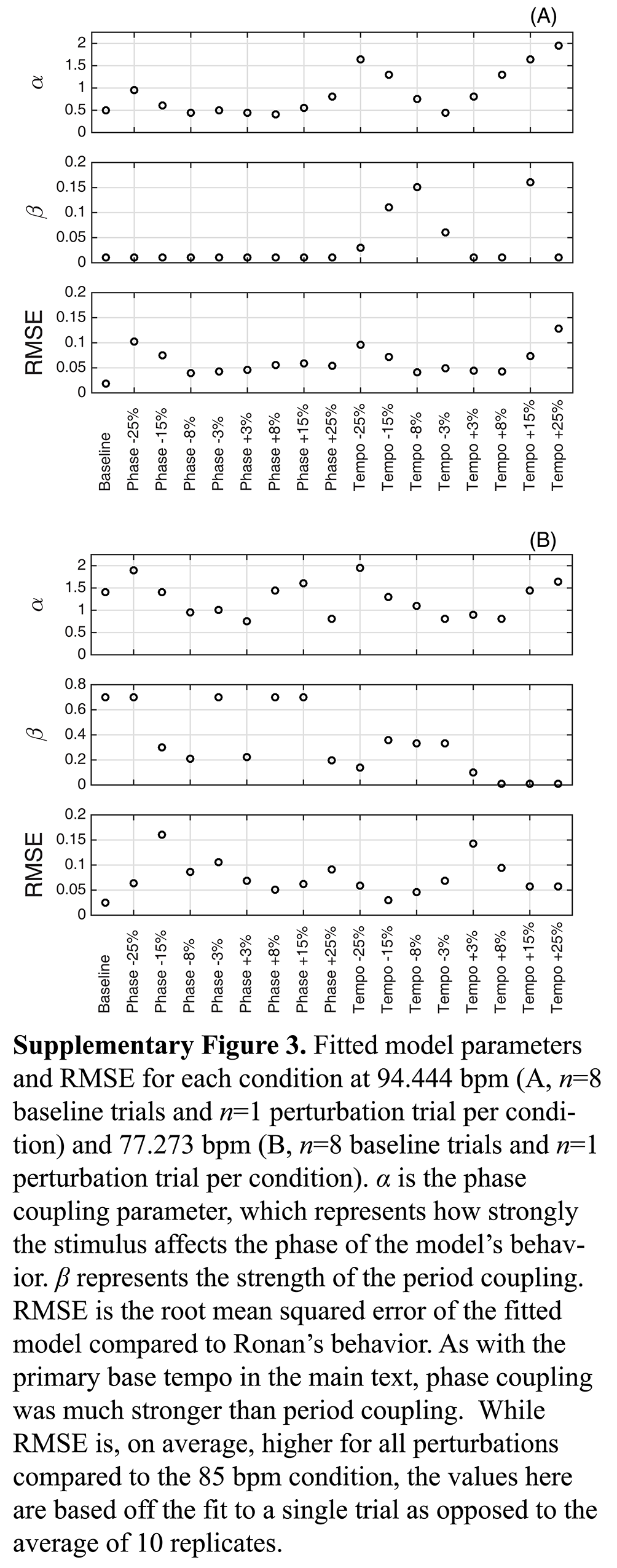

Supplement: Supplementary file 6 [file Image3.tif]
